# Exploring ddRAD sequencing data of tomato genotypes evaluated for the heat stress tolerance

**DOI:** 10.1016/j.dib.2024.110982

**Published:** 2024-10-01

**Authors:** Salvatore Graci, Amalia Barone

**Affiliations:** Department of Agricultural Sciences, University of Naples Federico II, 80055 Portici (NA), Italy

**Keywords:** *S. lycopersicum*, Abiotic stresses, High temperatures, Genomics, Polymorphisms, Reduced Representation Sequencing (RRS)

## Abstract

Climate change is a major concern for agricultural crops, and the selection of tolerant genotypes in response to abiotic stresses represents an important breeding strategy to reduce yield losses. In addition, the continuous development of new and more accurate high-throughput technologies for the analysis of DNA sequences is the key to improve biological understanding and application of biological knowledge. In the present work, 27 tomato genotypes already evaluated for their response under high temperature conditions were sequenced by using the ddRAD sequencing technology. The main goal was to provide genomic data useful for identifying candidate genes and variants to cope with current climate changes. Total genomic DNA was extracted from leaves and sequenced on the HiSeq2500 Illumina instrument. Raw reads of the dataset were processed using different bioinformatics tools to generate a Variant Calling Format (VCF) file. The availability of resources reporting polymorphisms among genomes of different genotypes provides a useful basis for studying tomato tolerance to current climate changes and can be used by researchers and breeders to investigate the molecular response mechanisms and develop new breeding programs, also aided by Marked Assisted Selection (MAS). The raw reads were deposited into SRA database (https://www.ncbi.nlm.nih.gov/sra/PRJNA1137563).

Specifications Table

**Table:** 

Subject	*Plant Science*
Specific subject area	*Investigation of ddRAD sequencing genomic data from tomato genotypes to identify suitable polymorphisms in response to abiotic stresses.*
Type of data	Raw fastq files Table Variant Calling Format (VCF) fIle Figure
Data collection	*The genomic data of the 27 tomato genotypes were obtained using ddRAD sequencing technology. Total genomic DNA was extracted from 100 mg of young leaf tissue from all the genotypes using the DNeasy plant mini kit (Qiagen, Hilden, Germany). For DNA sequencing, samples were used to prepare libraries for the ddRAD sequencing, as described in Peterson et al. 2012, PLoS ONE 7, e37135 with minor modifications. MboI and SphI enzymes were used for restriction digestion and fragments sequenced with the V4 chemistry paired end 125 bp mode on the HiSeq2500 Illumina instrument.*
Data source location	*Department of Agricultural Sciences, University of Naples Federico II, 80055 Portici (NA), Italy*
Data accessibility	Repository name: Sequence Read Archive (SRA-NCBI) Data identification number: BioProject: PRJNA1137563 Direct URL to data: https://www.ncbi.nlm.nih.gov/sra/PRJNA1137563 Instructions for accessing these data: The raw sequencing reads can be accessed and downloaded by visiting the direct URL.
Related research article	*Francesca, S., Vitale, L., Graci, S., Addonizio, M., Barone, A., & Rigano, M. M. (2024). Integrated physiological and genetic data reveal key-traits for heat tolerance in tomato. Plant Stress, 100555.*

## 1 Value of the Data

• The ddRAD sequencing data can be applied to perform genomic selection, gene/QTL mapping, linkage mapping, physical mapping, association mapping, genome-wide association studies (GWAS), identification of candidate genes, phylogenetic analyses and marker-assisted selection (MAS) for the investigation of tomato tolerance to abiotic stresses.
• These data represent a useful resource for researchers and breeders to investigate inheritance and genetics of complex traits, and identification of impactful variants for their application for marker-assisted breeding.
• The genomic data of the 27 tomato genotypes can be combined to predict heterozygous loci resulting in hybrids obtained by using these material as parental lines in breeding programs.

## 2 Background

The meteoric increase in sequencing technologies with next generation sequencing has dramatically changed the understanding of plant genomic variability. These tools have been widely applied to investigate the molecular response mechanisms to abiotic stresses of plants due to climate changes. Among these, heat stress can have adverse effects on plant morphology, physiology, and biochemistry during all stages of vegetative and reproductive development, thus comporting yield losses [1,2]. In this context, the double-digest Restriction enzyme-Associated DNA (ddRAD) sequencing based on the Reduced Representation Sequencing (RRS) technology provides an economic and feasible approach to obtain a portion of the whole sequence of large and repetitive plant genomes by employing two restriction enzymes with low-frequency and high-frequency cutter to digest DNA [[3], [4], [5], [6]]. ddRAD data can be used to perform genomic selection, QTL mapping, genome-wide association studies (GWAS) and identification of candidate genes [7]. This high-throughput platform represents a valuable tool for researchers and breeders in the investigation of molecular response mechanisms and selection of tolerant genotypes to cope with losses caused by abiotic stresses. The ddRAD sequencing platform was used to obtain genomics sequences of 27 tomato genotype evaluated for their response to high temperatures.

## 3 Data Description

The raw fastq reads of all the 27 tomato genotypes of the present study resulted from ddRAD sequencing were deposited into the Sequence Read Archive (SRA) database with the PRJNA1137563 BioProject ID (https://www.ncbi.nlm.nih.gov/sra/PRJNA1137563). Results of the reads processing are listed in Table 1.

**Table 1:** Results of the reads processing of each genotype. The number of reads resulted from sequencing, number of surviving reads obtained by the trimming step and the overall alignment rate of the mapping procedure are reported.

Genotype	Reads (n)	Trimmomatic - Surviving reads (n)	Bowtie2 – Overall alignment rate (%)
E7	1,232,565	1,131,107	97.60
E8	1,261,365	1,181,584	97.67
E17	1,085,550	1,013,981	97.42
E20	1,115,840	1,049,030	96.70
E23	329,975	304,103	96.61
E30	1,330,515	1,236,820	97.22
E36	1,892,722	1,758,527	97.09
E37	1,027,475	950,814	97.84
E40	1,332,121	1,253,466	97.28
E41	1,548,050	1,440,918	97.47
E42	1,140,754	1,062,495	95.92
E43	1,854,051	1,734,640	97.49
E45	844,507	798,409	97.31
E48	865,036	806,713	96.98
E53	1,011,532	941,581	97.81
E55	2,009,424	1,861,930	96.66
E75	1,341,756	1,238,443	96.38
E76	995,709	930,968	97.29
E107	1,078,373	1,010,265	97.60
E201	1,810,654	1,696,546	97.62
PDVIT	1,791,981	1,674,576	95.27
M82	1,771,544	1,666,978	96.91
MoneyMaker	1,531,477	1,419,470	97.33
LA2662	981,946	918,231	97.53
LA3120	1,604,292	1,488,258	97.14
DOCET	912,522	855,122	97.24
JAG8810	1,024,871	956,102	95.61

**Fig. 1:**
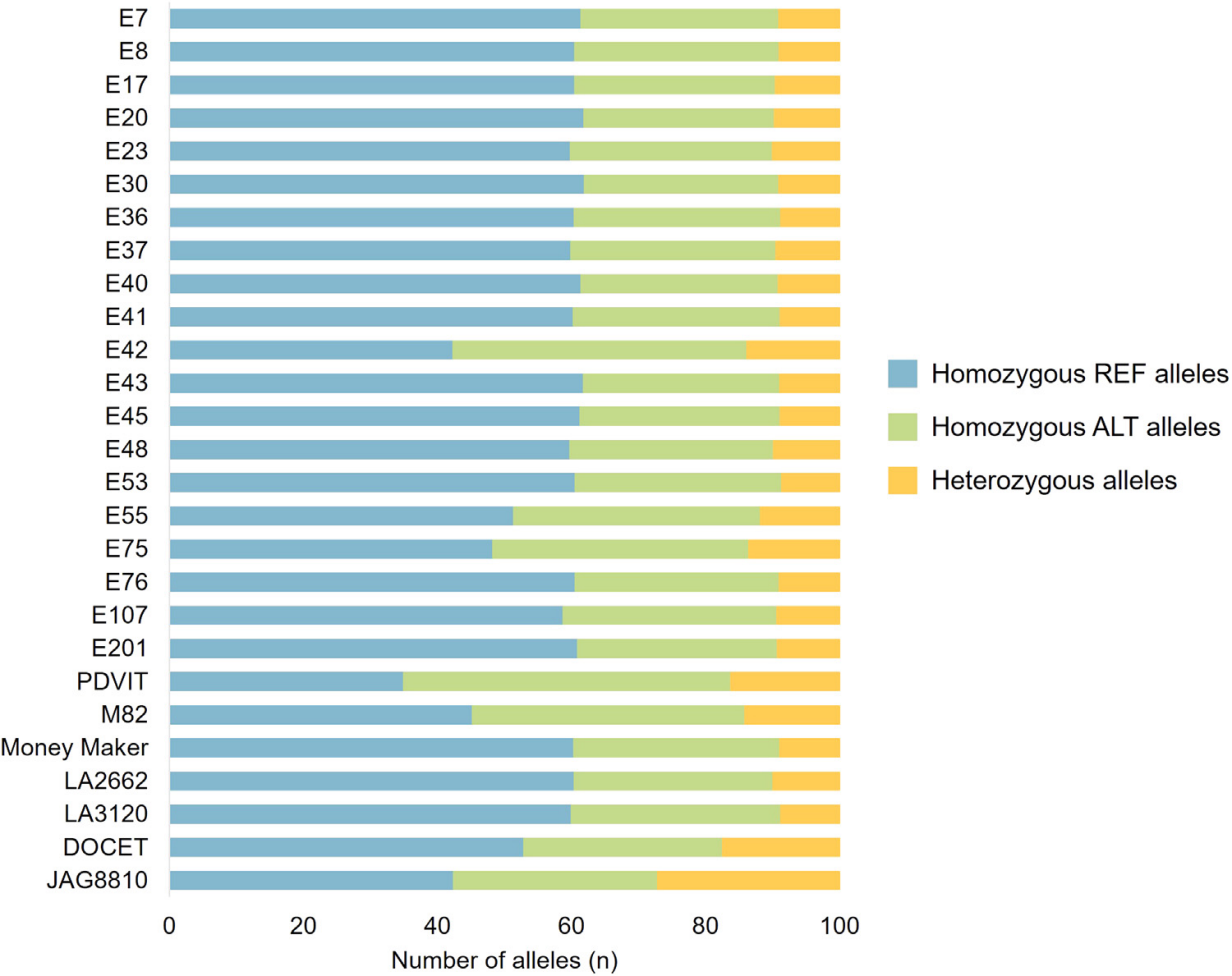
Graphical representation showing the number of homozygous reference-, homozygous alternative- and heterozygous alleles compared to Heinz *S. lycopersicum* reference genome of each genotype sequenced by ddRAD sequencing technology.

The Variant Calling Format (VCF) file is reported in Table S1 and evidenced a final dataset of 79,951 variants compared to the Heinz *S. lycopersicum* reference genome (SL4.0 version). Moreover, the number of homozygous and heterozygous variants of each genotype compared to the reference genome was also investigated and results are reported in Fig. 1.

Considering the homozygous variants respect to the Heinz reference genome, their distribution along the tomato genome was also assessed for all the twenty-seven genotypes focusing on each of the twelve chromosomes (Table 2).

**Table 2:** Distribution of the homozygous variants respect to the Heinz reference genome on each of the twelve tomato chromosomes.

Genotype	Variants (n)											
	Ch01	Ch02	Ch03	Ch04	Ch05	Ch06	Ch07	Ch08	Ch09	Ch10	Ch11	Ch12
E7	2,066	449	690	1,503	1,795	921	1,135	2,372	1,944	692	969	1,277
E8	2,076	447	709	1,522	1,785	881	1,132	2,370	1,851	602	976	2,171
E17	2,066	418	726	1,549	1,946	1,008	1,136	2,358	1,860	711	976	1,262
E20	2,098	397	694	1,464	1,773	917	1,136	2,352	1,940	640	793	1,148
E23	2,041	452	702	1,535	1,783	973	1,124	2,363	1,903	623	815	2,093
E30	2,130	419	743	1,576	1,754	919	1,149	2,366	1,955	623	798	1,209
E36	2,061	454	681	1,562	1,768	910	1,129	2,390	1,937	632	971	2,224
E37	2,054	454	708	1,566	1,775	907	1,140	2,347	1,923	614	941	2,170
E40	2,074	433	738	1,576	1,780	930	1,148	2,346	1,973	630	982	1,205
E41	2,065	417	688	1,537	1,770	907	1,138	2,379	1,937	739	962	2,165
E42	4,516	534	695	4,069	1,837	867	4,289	2,383	1,835	762	1,069	3,372
E43	2,094	442	679	1,471	1,773	916	1,125	2,376	1,945	624	954	1,294
E45	1,696	423	720	1,544	1,783	922	1,131	2,345	1,967	628	777	2,204
E48	2,047	445	696	1,542	1,777	908	1,134	2,361	1,924	618	933	2,134
E53	2,092	461	723	1,540	1,830	915	1,128	2,385	1,861	611	994	2,270
E55	2,077	505	847	4,022	1,778	1,419	1,342	2,413	2,608	833	819	2,266
E75	2,020	622	1,187	3,587	4,635	1,061	1,164	2,453	1,937	776	958	1,624
E76	2,100	454	738	1,582	1,780	916	1,153	2,361	1,947	607	770	2,291
E107	2,074	452	737	1,572	1,767	995	1,132	2,396	1,862	1,283	975	2,292
E201	1,743	439	698	1,580	1,859	856	1,091	2,338	1,915	739	1,057	1,647
PDVIT	2,265	741	804	4,647	5,933	1,121	1,240	2,373	3,871	629	2,722	3,421
M82	1,631	491	715	4,075	5,516	920	1,147	2,331	1,846	748	2,577	1,651
MoneyMaker	2,087	453	715	1,529	1,788	895	1,139	2,384	1,843	611	1,006	2,240
LA2662	1,622	412	670	1,450	1,790	858	1,094	2,351	1,863	731	1,078	2,217
LA3120	2,087	436	710	1,820	1,820	928	1,094	2,399	1,873	630	992	2,229
DOCET	1,587	385	681	1,488	1,980	872	1,134	2,310	1,830	684	762	2,287
JAG8810	1,591	409	637	1,629	1,958	958	1,105	2,315	1,811	754	1,135	2,279

## 4 Experimental Design, Materials and Methods

A Variant Calling Format (VCF) file including sequences data retrieved by using ddRAD technology on 27 tomato genotypes [[8], [9], [10], [11]] was investigated. The 27 tomato lines were selected from a wide tomato germplasm collection available at the Department of Agricultural Sciences of the University of Naples Federico II, which includes genotypes showing high variability in terms of fruit shape and quality, plant habitus and tolerance to biotic and abiotic stresses [12,13]. A detailed list of the genotyped material is reported in Table 3.

**Table 3:** List of the genotypes sequenced by using ddRAD sequencing technology. The code, the common name and the origin of the genotypes are reported.

Code	Common name	Origin
E7	Corbarino PC04	Italy
E8	Corbarino PC05	Italy
E17	Pantano Romanesco	Italy
E20	Pizzutello	Italy
E23	SanMarzano 1-38 SMEC	Italy
E30	Sel PC07	Italy
E36	Vesuvio Foglia Riccia	Italy
E37	Siccagno del Vesuvio	Italy
E40	GiaGiù	Italy
E41	Parmitanella	Italy
E42	PI15250	Italy
E43	Principe Borghese	Italy
E45	SM246	Italy
E48	Vesuvio2001	Italy
E53	LA0147	Honduras
E55	LA0358	Colombia
E75	Gold Nugget	USA
E76	Black Plum	USA
E107	E-L-19	Spain
E201	-	-
PDVIT	Cannellino Vitiello	Italy
M82	M82	USA
Money Maker	Money Maker	USA
LA2662	Saladette	-
LA3120	Malintka 101	-
DOCET F_1_	DOCET F_1_	Seminis
JAG8810 F_1_	JAG8810 F_1_	Seminis

Raw FASTQ files were quality-filtered and trimmed using Trimmomatic [14] v.0.39 (http://www.usadellab.org/cms/?page=trimmomatic) with default parameters. Paired trimmed reads were aligned with the *Solanum lycopersicum* reference genome (Tomato Genome version SL4.0, available at the Solgenomics Network, www.solgenomics.net) using Bowtie-2 [15] with default parameters. The resulting BAM files were sorted, de-duplicated and indexed with Samtools [16]. Finally, the variant calling step was performed by using BCFtools mpileup [17] with default parameters. Only the positions showing no missing data for all the 27 genotypes were considered to generate the final VCF file. Finally, it was filtered by using VCFtools [18] setting the parameters as follows: minQ = 10 and minimum mean of depth of coverage (min–mean DP) = 10.

## Limitations

The ddRAD sequencing analyses provide only a random reduced representation of the whole genome variability. Some polymorphisms in genes of interest may not be detected by the sequencing technology.

## Ethics Statement

The authors have read and follow the ethical requirements for publication in Data in Brief and confirm that the current work does not involve human subjects, animal experiments, or any data collected from social media platforms.

## Credit Author Statement

**Graci Salvatore**: Methodology, Software, Validation, Formal analysis, Investigation, Data curation, Writing – original draft, Visualization. **Barone Amalia**: Conceptualization, Writing – review & editing, Funding acquisition.

## Data Availability

ddRAD sequencing data of 27 tomato genotypes (Original data) (Sequence Read Archive (SRA-NCBI)).

## Appendix Supplementary materials

Image, application 1
